# Cardiovascular Risk and Hippocampal Thickness in Alzheimer's Disease

**DOI:** 10.1155/2013/108021

**Published:** 2013-10-21

**Authors:** Markus Donix, Maria Scharf, Kira Marschner, Annett Werner, Cathrin Sauer, Antje Gerner, Josef A. Nees, Shirin Meyer, Katharina L. Donix, Rüdiger Von Kummer, Vjera A. Holthoff

**Affiliations:** ^1^Department of Psychiatry and Psychotherapy, Division of Old Age Psychiatry and Cognitive Neuropsychiatry, University Hospital Carl Gustav Carus, Technische Universität Dresden, 01307 Dresden, Germany; ^2^German Center for Neurodegenerative Diseases (DZNE), 01307 Dresden, Germany; ^3^Department of Neuroradiology, University Hospital Carl Gustav Carus, Technische Universität Dresden, 01307 Dresden, Germany

## Abstract

Cardiovascular risk factors influence onset and progression of Alzheimer's disease. Among cognitively healthy people, changes in brain structure and function associated with high blood pressure, diabetes, or other vascular risks suggest differential regional susceptibility to neuronal damage. In patients with Alzheimer's disease, hippocampal and medial temporal lobe atrophy indicate early neuronal loss preferentially in key areas for learning and memory. We wanted to investigate whether this regional cortical thinning would be modulated by cardiovascular risk factors. We utilized high-resolution magnetic resonance imaging and a cortical unfolding technique to determine the cortical thickness of medial temporal subregions in 30 patients with Alzheimer's disease. Cardiovascular risk was assessed using a sex-specific multivariable risk score. Greater cardiovascular risk was associated with cortical thinning in the hippocampus CA2/3/dentate gyrus area but not other hippocampal and medial temporal subregions. APOE genotype, a family history of Alzheimer's disease, and age did not influence cortical thickness. Alzheimer's disease-related atrophy could mask the influence of genetic risk factors or age on regional cortical thickness in medial temporal lobe regions, whereas the impact of vascular risk factors remains detectable. This highlights the importance of cardiovascular disease prevention and treatment in patients with Alzheimer's disease.

## 1. Introduction

Patients with cardiovascular risk factors show an increased probability of developing cognitive decline and dementia [1, 2]. High blood pressure, diabetes, elevated cholesterol, and other vascular risks contribute to pathology resulting in cerebrovascular disease. However, studies have also demonstrated an association of these factors with Alzheimer's disease [3, 4]. Alzheimer's disease's neuropathological hallmarks, beta-amyloid plaques and neurofibrillary tangles, and vascular pathology frequently cooccur in demented patients. It is not yet obvious whether and how pathological changes associated with vascular risks are closely related to Alzheimer's disease etiology or whether they mainly mediate additional neuronal impairment ultimately contributing to the neurodegenerative disease's onset or clinical course. There is faster cognitive deterioration in Alzheimer's disease patients presenting with vascular risk factors [5], and, in contrast, treatment of such risks could slow this decline [6]. 

Damage of the blood-brain barrier with increased vascular permeability or high oxidative stress may be involved in both vascular dementia and Alzheimer's disease [7]. Therefore vascular disease could stimulate the development of Alzheimer's disease and vice versa [7]. Postmortem data show greater amyloid plaque burden in the hippocampus and in the neocortex of people that suffered from elevated systolic blood pressure [8]. Beta-amyloid peptide production could be increased in people with high blood pressure due to its vasoconstrictive properties [9]. Animal model data suggest a causal relationship between beta-amyloid plaque accumulation and hypertension, possibly through dysregulation of beta-amyloid trafficking [10]. Diabetes may contribute to cognitive decline and dementia via cerebrovascular changes such as atherosclerosis. However, insulin-degrading enzyme in the brain also regulates extracellular beta-amyloid levels [11]. Midlife cholesterol levels influence the development of Alzheimer's disease later in life, also possibly involving mechanisms other than the vascular pathway [12].

Cardiovascular risk contributes to regional brain structure changes in aging in the absence of cerebrovascular disease [13], which can be detected in vivo using magnetic resonance imaging (MRI). These data also suggest a higher vulnerability of specific brain regions when compared with others. Among patients with Alzheimer's disease, the earliest neuropathological changes can be found in the transentorhinal area before spreading to the hippocampus and subsequently to other brain regions [14]. Genetic risks, such as carrying the APOE-4 allele, contribute to brain structure changes [15] that may render people at higher risk of developing Alzheimer's disease. However, composite factors, for example, a positive family history of Alzheimer's disease, specifically acknowledge the possible influence of modifiable risk factors [16]. Raz and colleagues [17] demonstrated an accelerated age-related atrophy in the hippocampus but not in other brain regions among patients with high blood pressure, but it remains controversial whether or not hippocampal atrophy in aging is modulated by vascular risk [18]. 

The hippocampus is a heterogeneous area with subregions that might be differentially susceptible to the deleterious effects of vascular risk factors and resulting perfusion impairment [19, 20]. In this study we used high-resolution MRI and cortical unfolding image analysis [21] to investigate the relationship between subregional hippocampal thickness and cardiovascular risk factors in patients with Alzheimer's disease. We hypothesized that increased cardiovascular risk would be associated with cortical thinning in the hippocampus CA2/3/dentate gyrus area. In contrast to other hippocampal subfields, this region is affected by neuronal damage at later neuropathological stages of the disease [14], possibly allowing for in vivo detection of the subtle modulating effect of vascular risks on neuronal structure.

## 2. Methods 

### 2.1. Participants

We recruited 30 patients with Alzheimer's disease (mean age 71.4 years ± 4.9 years, Table 1) through our university hospital's memory clinic. These patients participate in cross-sectional and longitudinal investigations of biological and clinical parameters. Written informed consent was obtained and the university's ethics committee approved the study. We only recruited Alzheimer's disease patients who had the cognitive capacity for consent. This was established in a clinical evaluation by an independent psychiatrist. Alzheimer's disease patients met standard clinical criteria [22]. All patients underwent APOE genotyping, MRI scanning, and extensive neuropsychological assessments. The patients did not have a history of cerebrovascular disease, psychiatric or neurological disorders other than Alzheimer's disease, or any systemic disease possibly affecting brain function. Structural MRI evaluation and laboratory testing complemented the diagnostic procedures. We had 11 patients homozygous for the APOE-3 genotype in our sample and 19 patients carrying the APOE-4 risk allele including five homozygous carriers. There were no patients with the APOE-2/4 variant. All patients were right handed, on stable (>6 months) antidementive, for example, acetylcholinesterase inhibitor medication, and did not receive any other psychotropic medication. Investigators performing MRI scanning and cortical unfolding procedures were blinded for the patients' clinical and demographic information.

### 2.2. Vascular Risk Assessment

For the assessment of cardiovascular risk we used a sex-specific multivariable risk score [23]. This score is composed of several items (age, total and high-density lipoprotein cholesterol, systolic blood pressure, treatment for hypertension, smoking, and diabetes status) and has been developed in the Framingham Heart Study as a convenient tool to assess and to quantify cardiovascular risk and to guide preventive care [23]. 

### 2.3. MRI Scanning and Cortical Unfolding

**Figure 1:**
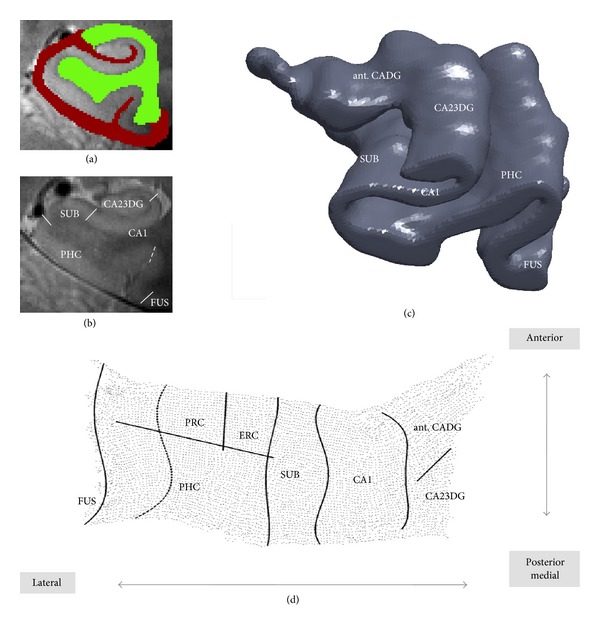
Cortical unfolding of the medial temporal lobe. Following manual segmentation of white matter and CSF (a), the resulting grey matter volume (c) is computationally unfolded and flattened ((d), right hemisphere). Boundaries between subregions are delineated on the original high-resolution MRI data (b) and later mathematically projected to flatmap space. Abbreviations: ant. CADG: anterior cornu ammonis fields and dentate gyrus, CA23DG: cornu ammonis fields 2,3 and dentate gyrus, CA1: CAfield 1, SUB: subiculum, ERC: entorhinal cortex, PRC: perirhinal cortex, PHC: parahippocampal cortex, FUS: fusiform cortex (boundary depicts the medial fusiform vertex).

Using a GE Signa HDxt 3-Tesla scanner (General Electric Health Care, Waukesha, Wisconsin) we obtained high-resolution oblique coronal T2-weighted fast-spin echo scans (repetition time: 5,200 msec; echo time: 105 msec; slice thickness: 3 mm; spacing: 0 mm; 19 slices; in-plane voxel size: 0.39 × 0.39 mm; field of view: 200 mm). Subsequent cortical unfolding procedures [21] result in a two-dimensional representation of the medial temporal lobe's gray matter, substantially improving the visibility of the convoluted medial temporal lobe cortex (Figure 1). Briefly, after manually masking white matter and CSF, gray matter is grown out in connected layers using a region-expansion algorithm. Because we maximized in-plane resolution, MRI data were interpolated by a factor of seven, in order to achieve an approximately isotropic voxel size. The gray-matter volume containing cornu ammonis fields 1 (CA1), CA2, 3, and the dentate gyrus (CA23DG), the subiculum (SUB), entorhinal (ERC), perirhinal (PRC), and parahippocampal cortices (PHC), and the fusiform gyrus (FUS) is then computationally unfolded based on metric multidimensional scaling algorithms. Boundaries between subregions are delineated on the original MRI sequence using histological and MRI atlases [24, 25] and mathematically projected to their flat map space coordinates. 

Consistent with our previous studies utilizing cortical unfolding, we report raw thickness data, which is also in line with different image analysis strategies that should be applied to cortical thickness measurements in contrast to volumetric data [26]. After confirming the normality of value distribution with the Kolmogorov-Smirnov test, we first estimated a multivariate general linear model, with APOE genotype and family history as between-group factors and cardiovascular risk score, MMSE score, and age as covariates. We used subregions as a within-group factor and conducted post hoc univariate tests across individual areas only after we established significance with the multivariate *F* tests in order to minimize spurious findings within the regions we can separate in our image analysis. Post hoc linear regression analyses were conducted to determine the influence of vascular risk and MMSE performance on regional cortical thickness.

## 3. Results

**Figure 2:**
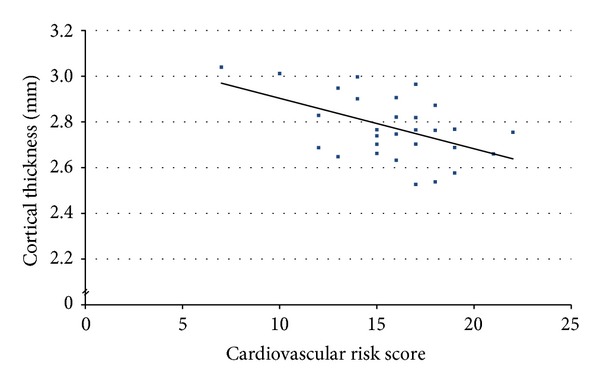
CA23DG thickness and cardiovascular risk. This figure shows that greater cardiovascular risk is associated with hippocampal thinning in the CA23DG region (ß = −0.5, *P* = 0.004) among Alzheimer's disease patients. This effect was not detectable in other medial temporal subregions investigated. We utilized a cardiovascular risk score, developed in the Framingham Heart Study [23], composed of the factors age, total and high-density lipoprotein cholesterol, systolic blood pressure, treatment for hypertension, smoking, and diabetes status.

For patient demographic and cognitive characterization, see Table 1. Utilizing a sex-specific multivariable risk score [23], our patients scored on average 15.3 points (SD 3.1, range 7–22). A high global risk (10-year risk >20%) of coronary, cerebrovascular, and peripheral arterial disease and heart failure [23] would be reflected in a score >17 points for women and >14 points for men. There were 15 patients with this high-risk profile among study participants. In our primary hippocampal region of interest (CA23DG), average cortical thickness across all patients was 2.78 millimeter (SD 0.14, range 2.52–3.04) in CA23DG. The multivariate general linear model revealed significant main effects for cardiovascular risk (*F* = 4.41, df = 7,14, *P* = 0.009) and MMSE score (*F* = 9.15, df = 7,14, *P* < 0.001) but not for APOE genotype, family history of Alzheimer's disease, and age. Linear regression showed that greater cardiovascular risk was associated with cortical thinning in the CA23DG region (ß = −0.5, *P* = 0.004, Figure 2) but not in CA1 or other medial temporal lobe subregions. Lower MMSE performance was associated with reduced cortical thickness in several subregions: ERC: ß = 0.4, *P* = 0.037, PRC: ß = 0.74, *P* < 0.001, PHC: ß = 0.6, *P* < 0.001, FUS: ß = 0.58, *P* < 0.001, and across all regions combined: ß = 0.59, *P* < 0.001. We did not detect hemispheric differences in the observed effects.

## 4. Discussion

We show an association between elevated cardiovascular risk and hippocampal thinning in our patients with Alzheimer's disease. This effect was restricted to the CA23DG region. APOE genotype, a first-degree family history of Alzheimer's disease, and age did not influence regional medial temporal lobe cortical thickness in our sample. With respect to age, this is particularly important since age itself is a component in the cardiovascular risk score we utilized [23]. It is possible that age effects on cortical thickness in this brain region are no longer obvious due to the substantial influence of disease-related atrophy. This is in line with disease severity, as reflected by the MMSE score, being associated with cortical thinning across several medial temporal subregions. 

Genetic risks, such as carrying the APOE-4 allele, contribute to cortical thinning in the entorhinal cortex and the subiculum in cognitively healthy people [15]. APOE-4-associated cortical thinning in the hippocampus CA1 field has been reported as well [16], whereas CA23DG cortical thickness was not modulated by APOE genotype [15, 16]. Among our patients with Alzheimer's disease we did not detect APOE effects on cortical thickness; however, others have found regionally selective APOE-4 effects on the hippocampal region [27]. Other variables, as reflected in a family history of Alzheimer's disease, influence hippocampal cortical thickness independently and through interaction with the APOE genotype [16]. Furthermore, APOE-4 allele dose modulates hippocampal volume in Alzheimer's disease patients [28]. The individual pattern of risk factors and their interactions could therefore contribute to some variability in brain imaging studies. Subtle APOE-4-associated changes on cortical structure may also become less consistently detectable in patients with a neurodegenerative disease. It is in line with the hypothesis that APOE is more important for the development rather than for the progression of Alzheimer's disease [29].

We cannot determine, using structural MRI, whether and how vascular risk factors themselves contribute to Alzheimer's disease development. The impact of blood pressure and blood sugar concentration elevation on beta-amyloid metabolism has been demonstrated in vitro and in animal models, pointing to possible pathways other than microvascular disease, such as dysregulation of beta-amyloid trafficking [10, 11] and blood-brain barrier damage [7]. Cardiovascular risk factors increase the risk of Alzheimer's disease [3, 4] and contribute to Alzheimer's disease progression [30]. Encouraging data show that treatment of such factors can reduce the conversion to Alzheimer's disease in patients with mild cognitive impairment [2]. Moreover, it was shown that the treatment of cardiovascular risk factors could slow cognitive decline in Alzheimer's disease even in the absence of obvious cerebrovascular disease [6]. The authors describe a progressive gradient of cognitive decline according to how many vascular risk factors were treated (all, some, or none), which suggests a causal relationship [6]. The association between antihypertensive treatment and the prevention of dementia and cognitive decline is well established, whereas the influence of treating hyperlipidemia on dementia risk is yet inconclusive, and positive effects may only be seen in specific populations [31, 32]. Lifestyle changes known to strengthen cardiovascular health, from Mediterranean diet to physical exercise, substantially contribute to cognitive fitness, lowering the risk of developing dementia [33]. 

Regionally specific brain structure changes shape our understanding of how a neurodegenerative disease develops or progresses. These characteristics guide research strategies, clinical diagnostic procedures, and the search for meaningful noninvasive biological markers. High-resolution MRI and innovative image analysis techniques make it possible to detect such brain structure changes in vivo, complementing other research modalities. Our MRI data suggest that, in Alzheimer's disease patients, CA23DG is more susceptible than other regions to vascular risk factor-associated changes. This would be in line with the neuropathological observation of the CA3 and dentate gyrus region being affected at later stages of Alzheimer's disease [14]. The CA23DG region's structural integrity could be relatively intact so that it would respond to the subtle modulating influence of vascular risks, whereas greater neuronal damage in other regions already exceeds this effect. In a postmortem study, Schwartz and colleagues [20] showed an increase in mean vessel density and variance in the hippocampus CA1 field of Alzheimer's disease patients, reflecting CA1 vulnerability to metabolic stress [19]. The CA3 field showed only milder vascular changes [20]. This could again indicate that strategies aimed at preventing the deleterious effects of cardiovascular risk factors might be beneficial for a region that is not already affected by extensive and permanent vascular pathology. 

Memory impairment is highly interrelated to hippocampal CA3 and dentate gyrus functioning through the region's essential role in pattern separation processes [34]. The main afferent input to this region is ERC neurons [25]. Pattern separation performance as well as ERC-CA3/dentate gyrus connectivity is already affected in normal aging [35]. There will be additional network changes due to ERC pathology-associated CA3/dentate gyrus deafferentation in Alzheimer's disease patients. It remains unknown how much vascular risk factors contribute to these network changes. Furthermore, Alzheimer's disease patients show impaired neurogenesis in the dentate gyrus [36], and, among other factors, vascular changes are possibly involved [37]. 

This study has several limitations. We did not examine healthy controls or other patient populations, which should be done in the future. Therefore, we cannot determine whether or not the association between cardiovascular risk and CA23DG thickness could be observed in individuals other than patients with Alzheimer's disease. Previous work suggests that cortical structure changes due to the presence of cardiovascular risk factors can be detected in various brain areas including the hippocampus among healthy people [13, 17]. Raz and colleagues [17] found age-associated hippocampal volume changes only among participants with a diagnosis of hypertension. This interaction could not be detected in adjacent brain regions, such as the entorhinal cortex, suggesting that areas within the medial temporal lobe show differential vulnerability to vascular risk factors. However, due to methodological differences, these data do not exclude the possibility that the risk factor-associated patterns of subregional hippocampal structure changes differ between Alzheimer's disease patients and nondemented subjects. Furthermore we are measuring cortical thickness, which does not allow direct comparisons with other studies investigating volumetric brain changes in Alzheimer's disease patients.

Age itself did not influence the association between cardiovascular risk and cortical thickness in our sample, but it is a contributing factor in the cardiovascular risk score we utilized [23]. Therefore a score value does not only indicate the presence of specific additional risk factors (such as hypertension or diabetes). Although we had a homogeneous distribution from low-risk to high-risk patients, we were not able to establish patient subgroups (e.g., subjects with no risk factors other than their age) due to sample size limitations. We also might not have enough power to detect cortical thickness changes associated with the APOE-4 allele. However, our previous investigations in healthy people differing in APOE genotype suggest that the absence of this effect is not mainly driven by sample size [15, 16]. Finally, it is important to underline that an association of elevated cardiovascular risk with cortical thinning in the hippocampus CA23DG region using in vivo MRI does not prove a causal relationship between these risk factors and Alzheimer's disease etiology or the disease's clinical course. Although the CA23DG region responds to the subtle influence of vascular risk factors and other regions do not, cortical thickness measures alone are not sufficient to show that the neuronal architecture in this region is more preserved until later stages of the disease [14]. CA fields 2,3, and the dentate gyrus are difficult to differentiate using in vivo MRI, which may influence comparability with postmortem data. 

## 5. Conclusions

Our data suggest a differential influence of cardiovascular risk on cortical thickness in hippocampal subregions among patients with Alzheimer's disease. Other well-known risk factors of the disease, such as APOE-4, a positive family history, and age, did not modulate hippocampal cortical thickness in these patients. This highlights the possible impact of modifiable risk factors in a brain region to be critically important for the clinical presentation of Alzheimer's disease patients. 

## Figures and Tables

**Table 1:** Demographic and cognitive characteristics.

Characteristic (range), mean ± SD	Alzheimer's disease patients (*N* = 30)
Age (years)	71.4 ± 4.9
Female sex (no.)	15
APOE-4 carriers (no.)	19
Positive family history (no.)	12
Minimental state examination (0–30)	21.7 ± 6.0
Alzheimer's disease assessment Scale-cognitive subscale (ADAS-Cog)	
Learning/memory	
Word recall (0–10)	6.48 ± 1.81
Word recognition (0–12)	5.87 ± 3.46
Remembering test instructions (0–5)	0.7 ± 1.42
Orientation (0–8)	2.47 ± 1.87
Language	
Commands (0–5)	0.8 ± 1.03
Spoken language (0–5)	1.07 ± 1.29
Naming objects and fingers (0–5)	0.63 ± 1.22
Word finding (0–5)	1.63 ± 1.54
Comprehension (0–5)	1.07 ± 1.08
Praxis	
Constructional praxis (0–5)	0.63 ± 0.62
Ideational praxis (0–5)	0.77 ± 1.31
Trailmaking test	
Part A (sec.)	83.19 ± 34.41
Part B (sec.)	201.91 ± 74.7
